# On the preconditioning of the primal form of TFOV-based image deblurring model

**DOI:** 10.1038/s41598-023-44511-x

**Published:** 2023-10-13

**Authors:** Junseok Kim, Shahabz Ahmad

**Affiliations:** 1 Department of Mathematics, Korea University, Seoul, South Korea; 2 ASSMS, Government College University, Lahore, Pakistan

**Keywords:** Applied mathematics, Computational science, Computer science

## Abstract

To address the staircasing problem in deblurred images generated by a simple total variation (TV) based model, one approach is to use the total fractional-order variation (TFOV) image deblurring model. However, the discretization of the Euler–Lagrange equations for the TFOV-based model results in a nonlinear ill-conditioned system, which adversely influences the performance of computational methods like Krylov subspace algorithms (e.g., Generalized Minimal Residual, Conjugate Gradient). To address this challenge, three novel preconditioned matrices are proposed to improve the conditioning of the primal model when using the conjugate gradient method. These matrices are designed based on circulant approximations of the matrix associated with blurring kernel. Experimental evaluations demonstrate the effectiveness of the proposed preconditioned matrices in enhancing the convergence and accuracy of the conjugate gradient method for solving the primal form of the TFOV-based image deblurring model. The results highlight the importance of appropriate preconditioning strategies in achieving robust and high-quality image deblurring using the TFOV approach.

## Introduction

Variational methods are an approach used in image deblurring in the last few decades, to restore sharpness and clarity to blurry images. They involve formulating an optimization problem that aims to find the best estimate of the original sharp image given the observed blurry image. In variational methods, following mathematical model is defined to describe the degradation process that caused the image blur.

1 $$\begin{aligned} z={{\textbf {K}}}u+\varepsilon , \end{aligned}$$

where *u* is an original image, *z* is a recorded image, $\varepsilon$ is the noise function, and

2 $$\begin{aligned} ({{\textbf {K}}}u)(x) = \int _{\Omega }k(x,y)u(y) ~dy,\quad x \in \Omega . \end{aligned}$$

If the blurring operator ${\textbf{K}}$ is given, then the corresponding approach is referred to as non-blind deconvolution^1–3^. However, when the blurring operator is unknown, the corresponding approach is referred to as blind deconvolution^4–7^. In this paper, our primary focus is on non-blind deconvolution. Here ${{\textbf {K}}}$ represents a first kind Fredholm-integral operator. Therefore, it is compact and the problem (1) becomes ill-posed^8–10^. Let $\Omega$ be a two-dimensional square domain. This model typically involves convolution with a blurring kernel that represents the blurring effect. The goal is to find the original image that, when convolved with the blurring kernel, closely matches the observed blurry image. The following optimization problem is formulated by constructing an objective function that consists of two terms: a data fidelity term (first term) and a regularization term (second term).

3 $$\begin{aligned} \underset{u}{min}\ \quad \left\{ T(u)=\frac{1}{2}\Vert {{\textbf {K}}} u - z \Vert ^2+\lambda J(u)\right\} . \end{aligned}$$

The data fidelity term measures the mismatch between the observed blurry image and the estimated sharp image after convolution with the blurring kernel. The regularization term *J*(*u*) encourages the restoration algorithm to produce visually desirable solutions by imposing certain constraints or promoting specific image properties.

One common regularization term used in variational deblurring methods is the total variation (TV) term^8,10–14^.

4 $$\begin{aligned} {TV}(u)=\int _{\Omega } \mid \nabla u \mid dx. \end{aligned}$$

The total variation of an image measures the amount of variation or changes in pixel intensities across the image. In a blurry image, neighboring pixels tend to have similar intensities, resulting in a low total variation. By promoting images with higher total variation, we can encourage the restoration of sharp edges and fine details. The idea behind TV regularization is to find an image that simultaneously fits the observed blurry image data and has a high total variation. This is achieved by solving an optimization problem that involves minimizing a cost function, which combines a fidelity term that measures the difference between the observed and reconstructed images, and a regularization term that quantifies the total variation. Minimizing the total variation encourages the reconstruction algorithm to produce an image with sharp transitions between regions of different intensities. It helps in removing blur and enhancing edges while preserving important image structures. By incorporating total variation regularization into the deblurring process, it is possible to obtain visually pleasing and sharper images, effectively reducing the impact of blur and noise in the original blurry image.

While TV regularization is a widely used method in image deblurring, it does have some drawbacks. One of the major drawbacks is staircase effect. TV regularization can introduce a “staircase effect” artifact, where edges appear as a series of steps rather than smooth transitions. This effect occurs because TV regularization promotes piecewise constant regions, resulting in a blocky appearance around edges instead of accurately representing their continuous nature.

Researchers continue to explore and develop alternative regularization techniques and algorithms to address these limitations and improve the quality of image deblurring methods. One of the alternative regularization is total fractional-order variation (TFOV)^8,15–25^ based regularization.

5 $$\begin{aligned} {TV}^{\alpha }(u)=\int _{\Omega } \mid \nabla ^{\alpha } u \mid dx. \end{aligned}$$

The TFOV regularization offers a powerful regularization approach for image deblurring problems, combining the advantages of edge preservation, flexibility, noise robustness, and reducing staircase effects. Its effectiveness has been demonstrated in numerous studies and has contributed to the advancement of image deblurring techniques. However, discretizing the TFOV-based model’s Euler–Lagrange (EL) equations results in a large nonlinear ill-conditioned system. To solve such systems efficiently is quite challenging for numerical methods. Even the powerful numerical algorithms like Krylov subspace methods (Generalized Minimal Residual (GMRES), Conjugate Gradient (CG) etc.) get slow convergence. One of the remedy for slow convergence is to use preconditioning.

Preconditioning is a technique that uses to transform a linear system of the form $Ax = b$ into another system to improve the spectral properties of the system matrix. A preconditioner is a matrix *P* such that this matrix is easy to invert and the preconditioned matrix $P^{-1}A$ has a good clustering behavior of the eigenvalues. Because rapid convergence is often associated with a clustered spectrum of $P^{-1}A$. In the Preconditioning technique, we solve the system $P^{-1}Ax=P^{-1}b$ instead of $Ax = b$ because the new system will converge rapidly when we use a suitable preconditioner. To apply the preconditioner matrix *P* within a Krylov subspace technique, we should calculate a matrix times a vector at each iteration. Hence, evaluating this product must be cheap. In the literature, several preconditioners^26–34^ are developed for the nonlinear systems. In this study, we consider the following non-linear system of equations

6 $$\begin{aligned} \Big (K_{h}^{*}K_h +\lambda [B_N (D_1(U)) \circ (B_N U)]+[(D_2(U) \circ (U B_N))] B_N \Big ) U = K_{h}^{*}Z \end{aligned}$$

This system is derived by discretizing the EL equations associated with the TFOV based image deblurring problem. The coefficient matrix of this system is of size $2N^2$ by $2N^2$, where *N* is the number of pixels. This matrix is non symmetric, ill-conditioned, dense and huge. These properties make the development of an effective computational method more challenging. We know that using direct methods for solving (6) requires $O(N^3)$ and hence they are not applicable here. For this system, iterative methods like Krylov subspace (GMRES, CG etc.) methods are applicable. However, their convergence is too slow because they are sensitive to the condition numbers. Hence, the idea of preconditioning is needed to accelerate the convergence of the Krylov subspace methods. In this study, we propose three circulant symmetric positive definite (SPD) preconditioners for system (6). The proposed preconditioners not only increase the convergence rate of numerical method but also contribute in the quality of deblurred images.

The manuscript makes the following contributions: (i) It introduces an efficient and fast algorithm for solving the TFOV-based image deblurring problem. (ii) It introduces three novel preconditioned circulant matrices to address the nonlinearity and ill-conditioned characteristics of the large system in the TFOV model. (iii) It offers an improved treatment for the computationally expensive TFOV regularization functional.

The remaining paper is organized into several sections as follows: section “The TFOV-model” describes the TFOV-based image deblurring model. The Euler–Lagrange equations of the TFOV-model are also discussed in this section. Section “Euler–Lagrange equations” presents the discretization of the TFOV-model and its matrix structure. Section “Discretization of the TFOV-model” introduces the numerical implementation. The proposed circulant preconditioners for the PCG (Preconditioned Conjugate Gradient) method are also explained in the same section. In section “Numerical solution algorithm”, the numerical experiments are presented. Section “Conclusion” presents conclusions.

## The TFOV-model

Let $$BV^{\alpha }(\Omega )$$ denote

$$\begin{aligned} BV^{\alpha }(\Omega ):=\left\{ u \in L^1(\Omega ) \vert TV^{\alpha }(u)< +\infty \right\} \end{aligned}$$

with the $\alpha$-BV norm $\vert \vert u \vert \vert _{BV^{\alpha }} =\vert \vert u \vert \vert _{L^1} + \int _\Omega \vert \nabla ^{\alpha }u \vert dx$, where $\alpha$ is the order of the fractional derivatives. $TV^{\alpha }$ is defined by

$$\begin{aligned} TV^{\alpha }(u)=\int _\Omega \vert \nabla ^{\alpha }u \vert dx:=\sup _{\phi \in T}\int _\Omega \left( -u~ div^{\alpha }\phi \right) dx, \end{aligned}$$

where $div^{\alpha }\phi =\frac{\partial ^{\alpha } \phi _1}{\partial x }+\frac{\partial ^{\alpha } \phi _2}{\partial y }$. $\frac{\partial ^{\alpha } \phi _1}{\partial x}$ and $\frac{\partial ^{\alpha } \phi _2}{\partial y}$ are the fractional-order derivative along the *x* and *y* directions, respectively. The space *T* denotes

$$\begin{aligned} T:=\left\{ \phi \in C^{\ell }_0(\Omega ,{\mathbb {R}}^2) \vert \phi (x) \vert \le 1,\quad \forall x \in \Omega \right\} \end{aligned}$$

where $\vert \phi (x) \vert =\sqrt{\Sigma _{i=1}^{2}\phi _i^2}$ and $C^{\ell }(\Omega ,{\mathbb {R}}^2)$ is the space of $\alpha$-order continuously differentiable functions. Hence, when the total fractional-order variation (TFOV) model is applied, the previous problem is transformed into the equivalent task of identifying a $u\in {BV}^{\alpha }(\Omega ) \cap L^2(\Omega )$ that minimizes the functional

7 $$\begin{aligned} \begin{aligned} F^{\alpha }(u)&= \frac{1}{2} \parallel {{\textbf {K}}}u -z \parallel ^2 + \lambda {TV}^{\alpha }_{\beta }(u) \end{aligned} \end{aligned}$$

where ${TV}^{\alpha }_{\beta }$ is the modified total fractional-order variation and defined by

8 $$\begin{aligned} {TV}^{\alpha }_{\beta }(u)(u)=\int _{\Omega } \sqrt{\mid \nabla ^{\alpha }u \mid ^2+\beta ^2} dx, \end{aligned}$$

where $|\nabla ^{\alpha }u|^2=(D_{x}^{\alpha }u)^2+(D_{y}^{\alpha }u)^2$ and $\beta$ is employed to make ${TV}^{\alpha }_{\beta }$ differentiable at zero. $D_{x}^{\alpha }$ and $D_{y}^{\alpha }$ are the fractional derivatives along the subscript directions. The existence and uniqueness of a minimizer for the problem (7) have been extensively investigated, as discussed in works such as^35,36^.

### Fractional-order derivatives

Various definitions of fractional-order derivatives have been proposed in the literature to describe such derivatives^37,38^. In this paper, we will present some of these definitions. For a comprehensive mathematical treatment, a fractional-order derivative is represented as a function operator denoted by ${D^{\alpha }}_{[a,x]}$. It is important to note that the order $\alpha$ satisfies the condition $0< n-1< \alpha < n$, where $n=[\alpha ]+1$ and $[\cdot ]$ is the greatest integer function.

**1. Definitions of the left- and right-sided Riemann–Liouville (RL) functional derivatives**:

9 $$\begin{aligned} {D^{\alpha }}_ {[a,x]} f(x)= \frac{1}{\Gamma (n-\alpha )} \left( \frac{d}{dx}\right) ^{n} \int _{a}^{x} (x-t)^{n-\alpha -1}f(t) dt \end{aligned}$$

and

10 $$\begin{aligned} {D^{\alpha }}_ {[x,b]} f(x)= \frac{1}{\Gamma (n-\alpha )} \left( {\frac{-d}{dx}}\right) ^{n} \int _{x}^{b} (t-x)^{n-\alpha -1}f(t) dt, \end{aligned}$$

where

$$\begin{aligned} \Gamma (z)=\int _{0}^{\infty } e^{-t} t^{z-1} dt. \end{aligned}$$

**2. Definitions of the left- and right-sided Grünwald–Letnikov (GL) functional derivatives**:

11 $$\begin{aligned} ^G D^{\alpha }_ {[a,x]} f(x)= \lim _{h \rightarrow 0} \frac{\Sigma _{j=0}^{[\frac{x-a}{h}]} (-1)^{j} C^{j}_{\alpha } f(x-jh)}{h^\alpha } \end{aligned}$$

and

12 $$\begin{aligned} ^G D^{\alpha }_ {[x,b]} f(x)= \lim _{h \rightarrow 0} \frac{\Sigma _{j=0}^{[\frac{b-x}{h}]} (-1)^{j} C^{j}_{\alpha } f(x+jh)}{h^\alpha }, \end{aligned}$$

where

13 $$\begin{aligned} C^{j}_{\alpha }=\frac{\alpha (\alpha -1) \cdots (\alpha -j+1)}{j!}. \end{aligned}$$

**3. Definitions of the left- and right-sided Caputo (C) functional derivatives**:

14 $$\begin{aligned} ^C D^{\alpha } _ {[a,x]} f(x)= \frac{1}{\Gamma (n-\alpha )} \int _{a}^{x} (x-t)^{n-\alpha -1}f^{(n)}(t) dt \end{aligned}$$

and

15 $$\begin{aligned} ^C D^{\alpha }_ {[x,b]} f(x)= \frac{(-1)^{n}}{\Gamma (n-\alpha )} \int _{x}^{b} (t-x)^{n-\alpha -1}f^{(n)}(t) dt, \end{aligned}$$

where $f^{(n)}$ is the *nth*-order derivative.

## Euler–Lagrange equations

For the functional (7) and $1<\alpha <2$, the Euler–Lagrange equations are as follows:

16 $$\begin{aligned} & {{\textbf {K}}}^* ( {{\textbf {K}}} u - z) + \lambda L_{\alpha }(u) u=0,~\text {on}~~ \Omega \\ & D^{\alpha -2} \left( \frac{\nabla ^{\alpha }u }{\sqrt{|\nabla ^{\alpha }u|^2+\beta ^2}} \right) \cdot \vec {\eta }= 0,~~D^{\alpha -1}\left( \frac{\nabla ^{\alpha }u }{\sqrt{|\nabla ^{\alpha }u|^2+\beta ^2}} \right) \cdot \vec {\eta }= 0,~\text {in}~ \partial \Omega . \end{aligned}$$

Here, ${{\textbf {K}}}^*$ is an adjoint operator and $L_{\alpha }(u)$ is given as

17 $$\begin{aligned} L_{\alpha }(u)w=(-1)^{n} \nabla ^{\alpha }.\left( \frac{\nabla ^{\alpha } w }{\sqrt{|\nabla ^{\alpha }u|^2+\beta ^2}} \right) . \end{aligned}$$

### Proof

From (7), we have

18 $$\begin{aligned} F^{\alpha } (u) = \frac{1}{2} \int _{\Omega }^{}({{\textbf {K}}} u - z)^2 dx + \lambda \int _{\Omega } \sqrt{\mid \nabla ^{\alpha }u \mid ^2+\beta ^2} dx, \end{aligned}$$

Let $\nu \in W^{\alpha }_1(\Omega )=\{v \in L^1(\Omega ): \Vert v\Vert _{W^{\alpha }_1 (\Omega )}=\int _{\Omega }|v|dx+\int _{\Omega }|\bigtriangledown v|dx<+\infty \}$. For $u \in W^{\alpha }_1(\Omega ) \subset BV^{\alpha }(\Omega )$, the Gateaux derivative of functional $F^{\alpha } (u)$ is as follows:

19 $$\begin{aligned} \frac{\partial F^{\alpha } (u) \nu }{\partial \nu } = lim_{t \rightarrow 0} \frac{ F^{\alpha }(u + t \nu ) - F^{\alpha }(u)}{t} \end{aligned}$$

$$\begin{aligned} = lim_{t \rightarrow 0} \frac{ G_1 (u + t \nu ) - G_1(u)}{t} + lim_{t \rightarrow 0} \frac{ G_2 (u + t \nu ) - G_2(u)}{t}, \end{aligned}$$

where $G_1 (u) = \frac{1}{2} \int _{\Omega }^{}({{\textbf {K}}} u - z)^2 dx$ and $G_2 (u) = \lambda \int _{\Omega } \sqrt{\mid \nabla ^{\alpha }u \mid ^2+\beta ^2} dx$. By applying the Taylor series, we have

20 $$\begin{aligned} \frac{\partial F^{\alpha } (u) \nu }{\partial \nu } = \int _{\Omega }^{}{{\textbf {K}}}^* ({{\textbf {K}}} u - z) \nu dx + \int _{\Omega }^{}({{\textbf {W}}}. \bigtriangledown ^{\alpha } \nu ) dx, \end{aligned}$$

where $${{\textbf {W}}} = {\tilde{\alpha }} \frac{\nabla ^{\alpha } u }{\sqrt{|\nabla ^{\alpha }u|^2+\beta ^2}}$$.

Applying $\alpha$-order integration by parts^35^, we get

21 $$\begin{aligned} \int _{\Omega }^{}({{\textbf {W}}}. \bigtriangledown ^{\alpha } \nu ) dx = (-1)^n \int _{\Omega }^{}(\nu ^C div^{\alpha } {{\textbf {W}}} ) dx \end{aligned}$$

$$\begin{aligned} - \sum _{j=0}^{n-1} (-1)^j \int _{0}^{1} D^{\alpha + j - n}_{[a,x]} W_1 \frac{\partial ^{n-j-1} \nu (x)}{\partial x^{n-j-1}_1} \Big |^{x_1 = 1}_{x_1 = 0} dx_2 - \sum _{j=0}^{n-1} (-1)^j \int _{0}^{1} D^{\alpha + j - n}_{[x,b]} W_2 \frac{\partial ^{n-j-1} \nu (x)}{\partial x^{n-j-1}_2} \Big |^{x_2 = 1}_{x_2 = 0} dx_1. \end{aligned}$$

Case-I: If $u(x) \Big |_{\partial \Omega } = b_1(x)$ and $\frac{\partial u(x)}{\partial n} \Big |_{\partial \Omega } = b_2(x)$, so $\Big ( u(x) + t \nu (x) \Big ) \Big |_{\partial \Omega } = b_1(x)$ and $\frac{\partial \Big ( u(x) + t \nu (x) \Big )}{\partial n} \Big |_{\partial \Omega } = b_2(x)$. Then, it suffices to choose $\nu \in {\mathscr {C}}^1_0 (\Omega , {\mathbb {R}} )$ and we have

$$\begin{aligned}{} & {} \frac{\partial ^i \nu (x) }{\partial n^i} \Big |_{\partial \Omega } = 0, \quad i = 0,1, \\{} & {} \quad \Rightarrow \frac{\partial ^{n-j-1} \nu (x) }{\partial x_1^{n-j-1}} \Big |_{x_1 = 0,1} = \frac{\partial ^{n-j-1} \nu (x) }{\partial x_2^{n-j-1}} \Big |_{x_2 = 0,1} = 0, \quad n-j-1 = 0,1. \end{aligned}$$

Hence, (20) reduces to (16).

Case-II: If $\nu \in W^{\alpha }_1 (\Omega )$, then

$$\begin{aligned} \frac{\partial ^{n-j-1} \nu (x) }{\partial x_1^{n-j-1}} \Big |_{x_1 = 0,1} \ne 0, \quad \frac{\partial ^{n-j-1} \nu (x) }{\partial x_2^{n-j-1}} \Big |_{x_2 = 0,1} \ne 0, \quad n-j-1 = 0,1. \end{aligned}$$

Therefore, the boundary terms in (3) can only become zero if

$$\begin{aligned}{} & {} D^{\alpha + j - n}_{[a,x]} W_1 \Big |_{x_1 = 0,1} = D^{\alpha + j - n}_{[x,b]} W_2 \Big |_{x_2 = 0,1} = 0 \\{} & {} \quad \Rightarrow D^{\alpha + j - n} {{\textbf {W}}}. \vec {\eta } = 0, \quad j = 0,1. \end{aligned}$$

The proof is now finished. $\square$

## Discretization of the TFOV-model

To discretize $\Omega =(0,1)\times (0,1)$, we divide it into $N^2$ a uniform grid, where *N* is a positive integer. Let $(x_k, y_l)$ for $k,l = 0, 1,\ldots , N + 1$ within $\Omega$^35,36^. Assuming that *u* has a homogeneous Dirichlet boundary condition and utilizing the shifted Gr$\ddot{\textrm{u}}$nwald approximation approach^39,40^, we consider the following derivative:

22 $$\begin{aligned} D^{\alpha } f(x_k,y_l)= & {} \frac{\delta _{0}^{\alpha } f(x_k, y_l)}{h^\alpha }+O(h)=\frac{1}{2} \left( \frac{\delta _{-}^{\alpha }f(x_k, y_l)}{h^\alpha }+\frac{\delta _{+}^{\alpha }f(x_k, y_l)}{h^\alpha }\right) +O(h)\nonumber \\= & {} \frac{1}{2 h^\alpha }\left( \sum _{j=0}^{k+1} \omega _{j}^{\alpha } f_{k-j+1}^{l}+\sum _{j=0}^{N-k+2} \omega _{j}^{\alpha } f_{k+j-1}^{l}\right) + O(h) \end{aligned}$$

where $f_{s}^{l}=f_{s,l}$ and $$\omega _{j}^{\alpha }=(-1)^{j} \begin{pmatrix} \alpha \\ j \end{pmatrix} j=0,1,\ldots ,N$$ and $\omega _{0}^{\alpha }=1,~\omega _{j}^{\alpha }=(1-\frac{1+\alpha }{j})\omega _{j-1}^{\alpha }$ for $j>0$. By using the homogeneous boundary condition, we can obtain the following expression:

$$\begin{aligned} & \left[ \begin{array}{ccccc} \delta _{0}^{\alpha } f(x_1, y_l)\\ \delta _{0}^{\alpha } f(x_2, y_l)\\ \vdots \\ \vdots \\ \delta _{0}^{\alpha } f(x_N, y_l) \\ \end{array} \right] \\ {}&\quad = \underbrace{ \frac{1}{2 h^\alpha }\left[ \begin{array}{ccccc} 2 {\omega _{1}}^{\alpha } &{}\quad {\omega _{0}}^{\alpha }+{\omega _{2}}^{\alpha }&{}\quad {\omega _{3}}^{\alpha } &{}\quad \ldots &{}\quad {\omega _{N }}^{\alpha } \\ {\omega _{0}}^{\alpha }+{\omega _{2}}^{\alpha }&{}\quad 2 {\omega _{1}}^{\alpha } &{}\quad \ddots &{}\quad \ddots &{}\quad \vdots \\ {\omega _{3}}^{\alpha } &{}\quad \ddots &{}\quad \ddots &{}\quad \ddots &{}\quad {\omega _{3}}^{\alpha }\\ \vdots &{}\quad \ddots &{}\quad \ddots &{}\quad 2 {\omega _{1}}^{\alpha } &{}\quad {\omega _{0}}^{\alpha }+{\omega _{2}}^{\alpha } \\ {\omega _{N}}^{\alpha } &{}\quad \ldots &{}\quad {\omega _{3}}^{\alpha } &{}\quad {\omega _{0}}^{\alpha }+{\omega _{2}}^{\alpha } &{}\quad 2 {\omega _{1}}^{\alpha } \end{array} \right] }_{\hbox { }\ {B^{\alpha }}_{N}} \left[ \begin{array}{ccccc} f_{1}^{l}\\ f_{2}^{l}\\ \vdots \\ \vdots \\ f_{N}^{l} \\ \end{array} \right] . \end{aligned}$$

Now, we have the following properties:

1. $${\omega _{0}}^{\alpha }=1,~{\omega _{1}}^{\alpha }=-\alpha <0,~~1 \ge {\omega _{2}}^{\alpha } \ge {\omega _{3}}^{\alpha } \ge \cdots \ge 0.$$
2. $\sum _{k=0}^{\infty } {\omega _{k}}^{\alpha }=0,~~\sum _{k=0}^{m} {\omega _{k}}^{\alpha }\le 0$. Hence, using the Gershgorin circle theorem, we can obtain ${B^{\alpha }}_{N}$ which is a symmetric and negative definite Toeplitz matrix. We define the solution matrix at (*khx*, *lhy*), where $k,l = 1,\ldots , N$. The ordered solution vector of *U* is represented as $\vec {u} \in {\mathbb {R}}^{N^2 \times 1}$. The discrete version of differentiation for an $\alpha$-order derivative is given as
  23 $$\begin{aligned} {u_{x}}^{\alpha }=(I_N \otimes {B^{\alpha }}_{N})\vec {u}={B_{x}}^{\alpha } \vec {u} \end{aligned}$$

Similarly, we have

24 $$\begin{aligned} {u_{y}}^{\alpha }=({B^{\alpha }}_{N} \otimes I_N)\vec {u}={B_{y}}^{\alpha } \vec {u}, \end{aligned}$$

where ${u_{x}}^{\alpha }=({u_{11}}^{\alpha }, \ldots ,{u_{NN}}^{\alpha } )^T,~~~~{u_{y}}^{\alpha }=({u_{11}}^{\alpha }, \ldots ,{u_{NN}}^{\alpha } )^T,$ $\vec {u} =(u_{11}, u_{12},\ldots ,u_{NN})$, and $\otimes$ denotes the Kronecker product^38,41^. Now, if we use a finite difference scheme and the discrete fractional derivative shown above, then (16) and (17) lead to the following primal system

25 $$\begin{aligned} (K_{h}^{*}K_h +\lambda L^{\alpha }(U^m) ) U^{m+1} = K_{h}^{*}Z, \quad m=0,1,2\ldots ,N_F, \end{aligned}$$

Let $N_F$ be the number of Fixed Point Iterations and $K_h$ be a matrix satisfying

26 $$\begin{aligned} ({{\textbf {K}}} u)(x_i,y_j)\approx [K_h U]_{ij},~~i,j=1,2,\ldots ,N, \end{aligned}$$

where $[K_h U]_{ij,lm}=h^2 k (x_i-x_j, y_l-y_m)$. By utilizing the lexicographical order, the matrix $K_h$ is structured as a block Toeplitz with Toeplitz block matrix. The discrete numerical method for the matrix ${L^{\alpha }}_h (U^m)$ is given by:

27 $$\begin{aligned} \begin{aligned} \left( L^{\alpha }(U^m)\right) U^{m+1}=[B_N (D_1(U^m)) \circ (B_N U^{m+1})]+[(D_2(U^m) \circ (U^{m+1} B_N))] B_N. \end{aligned} \end{aligned}$$

Here, $\circ$ represents the pointwise multiplication operation, and *m* corresponds to the *m*-th Fixed Point Iteration. The matrix *U* is obtained by reshaping the vector *u* into an $N \times N$ matrix. $D_1(U^m)$ and $D_2(U^m)$ are diagonal matrices consisting of the element-wise reciprocals of the non-zero matrices ${B_{x}}^{\alpha }(U^m)$ and ${B_{y}}^{\alpha }(U^m)$, respectively.

We have the following total variation (TV)-based image deblurring method.

28 $$\begin{aligned} (K_{h}^{*}K_h +\lambda L^{TV}_h (U^m) ) U^{m+1} = K_{h}^{*}Z, \quad m=0,1,2\ldots ,N_F, \end{aligned}$$

where

29 $$\begin{aligned} L^{TV}_h(U^m) = G_h^* H_h^{-1}(U^m) G_h. \end{aligned}$$

Here, more details about the term $L^{TV}_h(U^m)$ can be found in^10^. The structure of matrix $B_h$ is as follows:

$$\begin{aligned}G_h=\frac{1}{h} \begin{bmatrix} G_1 \\ G_2 \end{bmatrix} \end{aligned}$$

where both $G_1$ and $G_2$ are of size $N(N - 1) \times N^2$ , and

$$\begin{aligned} G_1 = F \otimes {\tilde{I}} \quad \text{ and } \quad G_2 = {\tilde{I}} \otimes F. \end{aligned}$$

$$\begin{aligned}F = \begin{bmatrix} 1 &{}\quad -1 &{} &{} &{} &{} \\ &{}\quad 1 &{}\quad -1 &{} &{} &{} \\ &{} &{}\quad \ddots &{}\quad \ddots &{} &{} \\ &{} &{} &{}\quad \ddots &{}\quad -1 &{} \\ &{} &{} &{} &{}\quad 1 &{}\quad -1 \end{bmatrix},\end{aligned}$$

is a matrix of size $(N - 1) \times N$. The matrix $H_h$ is a diagonal matrix, which are calculated by discretizing $\sqrt{\left| \nabla u\right| ^2+\beta ^2}$. It possesses the following structure:

$$\begin{aligned}H_h= \begin{bmatrix} H^x &{}\quad 0 \\ 0 &{}\quad H^y \end{bmatrix}, \end{aligned}$$

where $H^x$ and $H^y$ are of sizes $(N - 1) \times N$ and $N \times (N - 1)$, respectively.

## Numerical solution algorithm

We describe the algorithms for solving the TFOV-based linear system (25). Before delving into the details, we present several essential properties of (25).

1. The Hessian matrix $K^*_h K_h + \lambda L^{\alpha }_h(U^m)$ is extremely large in practical applications. When the value of $\lambda$ is small, $K^*_h K_h + \lambda L^{\alpha }_h(U^m)$ becomes highly ill-conditioned. This is mainly due to the clustering of eigenvalues of ${{\textbf {K}}}$ around zero^10^.
2. $K^*_hK_h$ is symmetric positive definite. Despite $K^*_hK_h$ is dense, ${{\textbf {K}}}$ has the translation invariant property, which allows the application of the FFT method to compute $K^*_hK_h u$ in just *O*(*nlogn*) operations^10^.
3. In the TFOV model (25), the fractional matrix $L_{h}^{\alpha }(U^m)$ is dense, resulting in an expensive matrix-vector multiplication. On the other hand, in the TV model (28), the non-fractional matrix $L^{TV}_{h}(U^m)$ is sparse.
4. $L_{h}^{\alpha }(U^m)$ is symmetric positive semidefinite^10^. Consequently, the system (25) is symmetric positive definite.

### Preconditioned matrices

The conjugate gradient (CG) algorithm is an appropriate iterative algorithm for solving the system (25). The CG algorithm is an iterative algorithm commonly used to solve systems of linear equations. It is particularly well-suited for large, sparse, and symmetric positive definite matrices. The CG algorithm iteratively finds the solution by minimizing the residual error in each iteration along conjugate directions. It does not require the explicit storage of the entire matrix, making it memory-efficient for large-scale problems. The CG algorithm achieves convergence in a number of iterations equal to the size of the problem, making it an efficient solver. However, the CG algorithm may converge slowly for ill-conditioned matrices, so preconditioned conjugate gradient (PCG) algorithm are often employed to improve convergence speed. In order to achieve an efficient solution, a preconditioning matrix having SPD property is required. In this context, we introduce two SPD circulant preconditioning matrices. The first one is denoted as $P_1$, and it is defined as follows:

30 $$\begin{aligned} P_1 = \lambda \tilde{K^*_h}\tilde{K_h} + \gamma I_h, \end{aligned}$$

where $\gamma >0$, $\tilde{K_h}$ is an approximate circulant form of $K_h$, and $I_h$ is an identity matrix. The second preconditioning matrix $P_2$ is;

31 $$\begin{aligned} P_2 = \lambda \tilde{K^*_h}\tilde{K_h} + \gamma diag L^{TV}_h(U^m), \end{aligned}$$

where $diag(L^{TV}_h(U^m))$ is a diagonal matrix having entries from $L^{TV}_h(U^m)$ (29). The third preconditioning matrix $P_3$ is a product type preconditioner.

32 $$\begin{aligned} P_3 = (\lambda \tilde{K^*_h}\tilde{K_h} + \gamma I_h)^{1/2}\Big (\gamma diag(L^{TV}_h(U^m)) + I_h\Big )(\lambda \tilde{K^*_h}\tilde{K_h} + \gamma I_h)^{1/2}, \end{aligned}$$

The preconditioned matrices $P_1$, $P_2$, and $P_3$ are designed based on the concept of “operator splitting” techniques^10,42^. During the application of PCG to solve (25), the inversion of the preconditioner matrices ($P_1$, $P_2$, and $P_3$) becomes necessary. The inversion of $P_1$ and $P_2$ can be easily performed since their second terms are sparse matrices, and the inversion of $\tilde{K^*_h}\tilde{K_h}$ requires only $O(n \log n)$ floating-point operations using the FFTs, as explained in detail in^10^. The inversion of the preconditioning matrix $P_3$ involves inverting its middle term, $\gamma \text {diag}(L^{TV}_h(U^m)) + I_h$. Since this middle term is a sparse matrix, its inversion can be done straightforwardly as well.

For the inversion of $(\lambda \tilde{K^*_h}\tilde{K_h} + \gamma I_h)^{1/2}$, we also require only $O(n \log n)$ floating-point operations using the FFTs. The PCG method is summarized as follows.
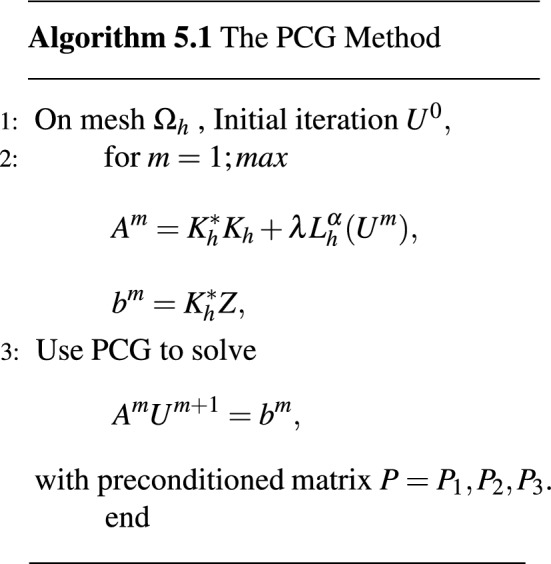


Next, let the eigenvalues of $K^*_h K_h$ and $L_h^{\alpha }(U^m)$ be $\mu _i^K$ and $\mu _i^{L^{\alpha } }$, respectively, such that $\mu _i^K \downarrow 0$ and $\mu _i^{L^\alpha } \uparrow \infty$. So the eigenvalues of $P^{-1}_1{\bar{A}}$ and $P^{-1}_2{\bar{A}}$ are

33 $$\begin{aligned} \eta ^1_i = \frac{\mu _i^K + \lambda \mu _i^{L^{\alpha }}}{\lambda \mu _i^K + \gamma } \quad \text{ and } \quad \eta ^2_i = \frac{\mu _i^K + \lambda \mu _i^{L^{\alpha }}}{\lambda \mu _i^K + \gamma \mu _i^{L^{TV}} }, \end{aligned}$$

respectively. Here $\mu _i^{L^{TV}}$ are eigenvalues of $L^{TV}_h(U^m)$. The ${\bar{A}} = K^*_h K_h +\lambda L^{\alpha }_h(U^m)$ is the Hessian matrix of (25). It is evident that $1 \le \mu _i^{L^{TV}} \le \mu _i^{L^\alpha }$. Then

34 $$\begin{aligned} \eta ^1_i \ge \frac{\mu _i^K + \lambda \mu _i^{L^{\alpha }}}{\lambda \mu _i^K + \gamma \mu _i^{L^{\alpha }} } \quad \text{ and } \quad \eta ^2_i \ge \frac{\mu _i^K + \lambda \mu _i^{L^{\alpha }}}{\lambda \mu _i^K + \gamma \mu _i^{L^{\alpha }} }, \end{aligned}$$

Clearly, for $$\gamma \equiv \lambda$$,

$$\begin{aligned} \eta _i^1, \eta _i^2 \rightarrow 1 \quad \text{ as } \quad i \rightarrow \infty . \end{aligned}$$

Hence, for $\gamma \equiv \lambda$, $P^{-1}_1{\bar{A}}$ and $P^{-1}_2{\bar{A}}$ exhibit better spectrum when compared with the Hessian matrix ${\bar{A}}$. Now, let eigenvalues of $P^{-1}_3{\bar{A}}$ be

35 $$\begin{aligned}{} & {} \eta ^3_i = \frac{\mu _i^K + \lambda \mu _i^{L^{\alpha }}}{(\lambda \mu _i^K + \gamma )(\gamma \mu _i^{L^{TV}} + 1)} \end{aligned}$$

36 $$\begin{aligned}{} & {} \ge \frac{\mu _i^K + \lambda \mu _i^{L^{\alpha }}}{(\lambda \mu _i^K + \gamma )(\gamma \mu _i^{L^{\alpha }} + 1)}. \end{aligned}$$

37 $$\begin{aligned}{} & {} \eta ^3_i \rightarrow 1, i \rightarrow \infty \quad \text{ for } \quad \gamma \equiv \lambda . \end{aligned}$$

Therefore, $P^{-1}_3{\bar{A}}$ exhibits better spectrum when compared with ${\bar{A}}$.

Preconditioning techniques can help mitigate issues like noise amplification and ringing artifacts that are often encountered in deblurring processes. By carefully designing the preconditioner, the optimization process can become more stable and effective, leading to better convergence towards a higher quality deblurred image. The preconditioners we introduce serve a dual purpose: they not only address the ill-posed characteristics of the problem but also aid in the retrieval of high-frequency intricacies within the deblurred images. This effect becomes evident through the numerical illustrations we provide.

### Numerical examples

**Figure 1:**
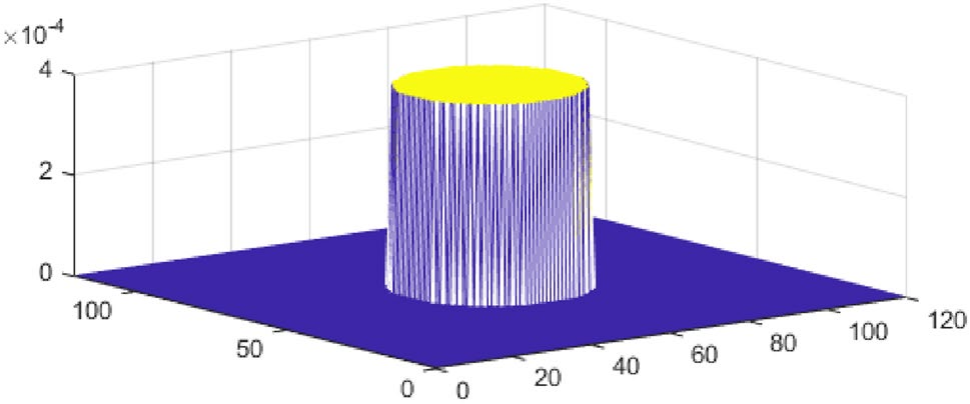
Schematic illustration of the blurring kernel.

Three numerical examples for the TFOV-based image deblurring problem are now presented. Various values of *N* were employed, resulting in a system with $N^2$ unknowns. MATLAB program, running on an Intel(R) Core(TM) i7-4510U CPU @ 2.60 GHz, was used for numerical computations and to obtain the numerical results. The evaluation of the deblurred images’ quality was conducted using PSNR (Peak Signal to Noise Ratio) and SSIM (Structured Similarity Index Measure). The $ke_{-}gen (N, r, \sigma )$ kernel^43–45^ was employed for numerical calculations. Figure 1 shows schematic illustration of the $ke_{-}gen (120, 40, 4)$ kernel.

**Initial guess and parameters** The works by^20,35,46–48^ delve into the intricate details of automatically selecting parameter $\lambda$, a topic that goes beyond the scope of this current paper. The careful choice of value for $\lambda$ holds paramount importance in effectively eliminating both blur and noise. It is advised to avoid extreme values, whether they are exceedingly large or exceptionally small. In the context of the TFOV-based model, the range deemed optimal for $\lambda$ lies between 1e−3 and 1e−7. As for the optimal range encompassing $\alpha$ and $\beta$, we conducted computational experiments employing the cameraman image. Our observations indicate that the most suitable range for $\alpha$ is situated between 1.1 and 1.5, while the optimal realm for $\beta$ spans from 0.6 to 1. The results of these computations are outlined in Tables 1 and 2. It is evident from our experiments that, for lower values of $\alpha$, higher values of $\beta$ are conducive. In our specific experimental setup, we made the deliberate selections of $\alpha = 1.4$, $\lambda = 1e$-6, and $\beta = 1$ to advance our research.

**Table 1:** PSNR against different value of $\alpha$ with fixed $\beta = 1$.

$\alpha$	TFOV (CG)	$P_1$CG	$P_2$CG	$P_3$CG
1	36.7328	36.5562	36.8945	36.6277
1.1	38.1952	38.8057	37.8956	37.9956
1.2	38.2259	38.7512	37.2565	37.9951
1.3	38.8015	38.2450	37.8929	38.0054
1.4	38.8061	38.8057	37.5909	37.9954
1.5	37.9924	37.2562	37.1685	37.9951
1.6	36.9575	36.5622	37.1121	36.9654
1.7	36.0059	36.5603	36.1923	36.2531
1.8	36.9583	36.2615	36.2513	36.1295
1.9	36.5666	36.8712	36.2412	36.1124
2	35.8212	36.1285	35.2655	36.1073

**Table 2:** PSNR against different value of $\beta$ with fixed $\alpha = 1.4$.

$\beta$	TFOV (CG)	$P_1$CG	$P_2$CG	$P_3$CG
0.1	36.5421	36.2451	36.4785	36.7541
0.2	36.2561	37.2561	37.2856	37.8452
0.3	36.2589	36.5892	37.9632	36.9856
0.4	37.2567	36.7542	36.9542	36.5727
0.5	37.8941	36.5896	36.4752	36.8555
0.6	38.5689	38.5612	37.4751	37.2568
0.7	38.1533	38.8561	37.8523	37.9865
0.8	38.5894	38.8621	37.4512	37.9799
0.9	38.8421	38.8432	37.9625	37.1985
1	38.8061	38.5412	37.5909	37.9954

#### Example 1

**Figure 2:**
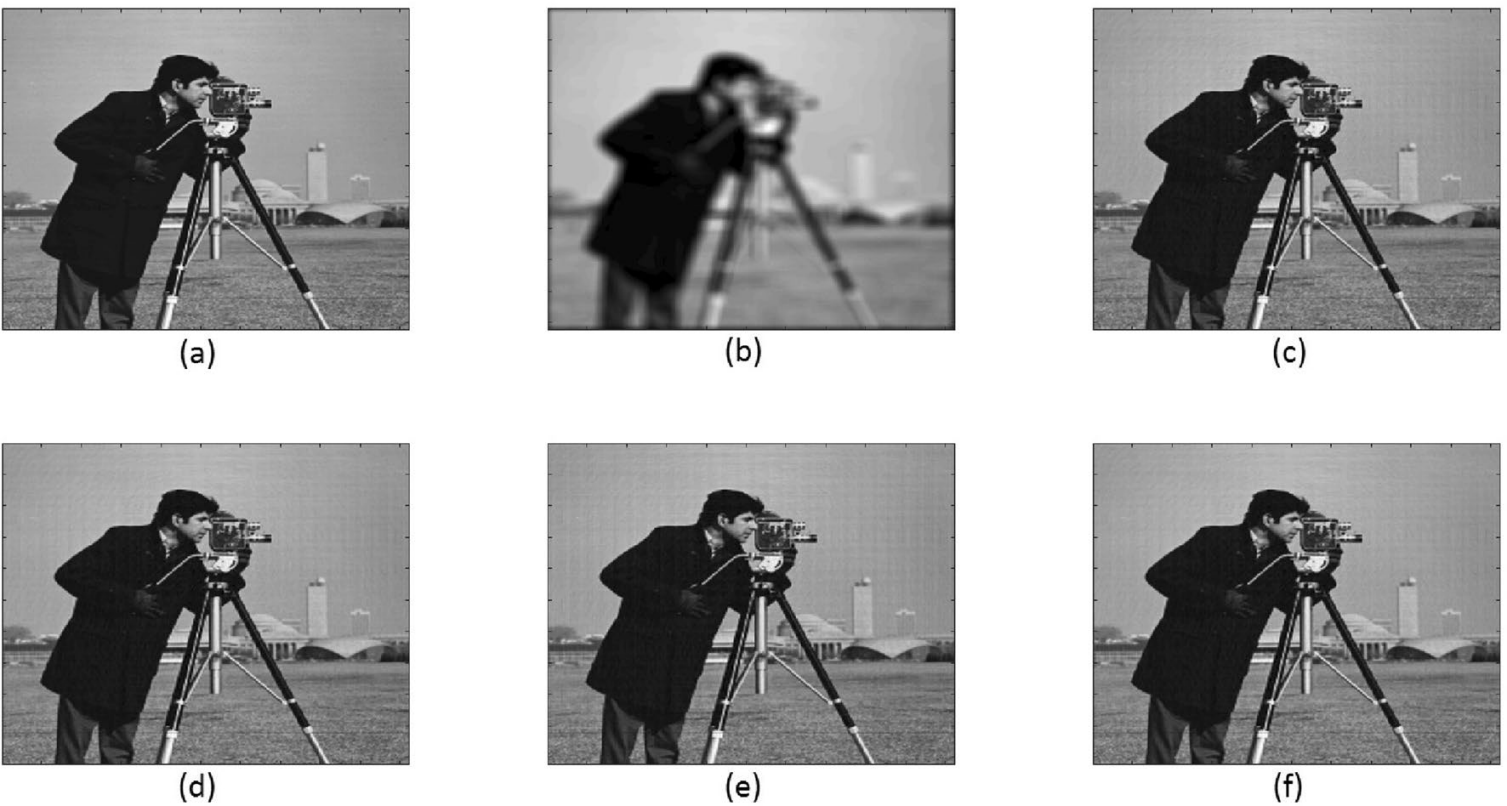
Cameraman image: (**a**) exact and (**b**) blurry images; (**c**–**f**) deblurred images by TFOV-based CG, $P_1$CG with $\gamma = 1e{-}3$, $P_2$CG with $\gamma = 1e{-}4$, and $P_3$CG with $\gamma = 1e{-}5$, respectively.

**Figure 3:**
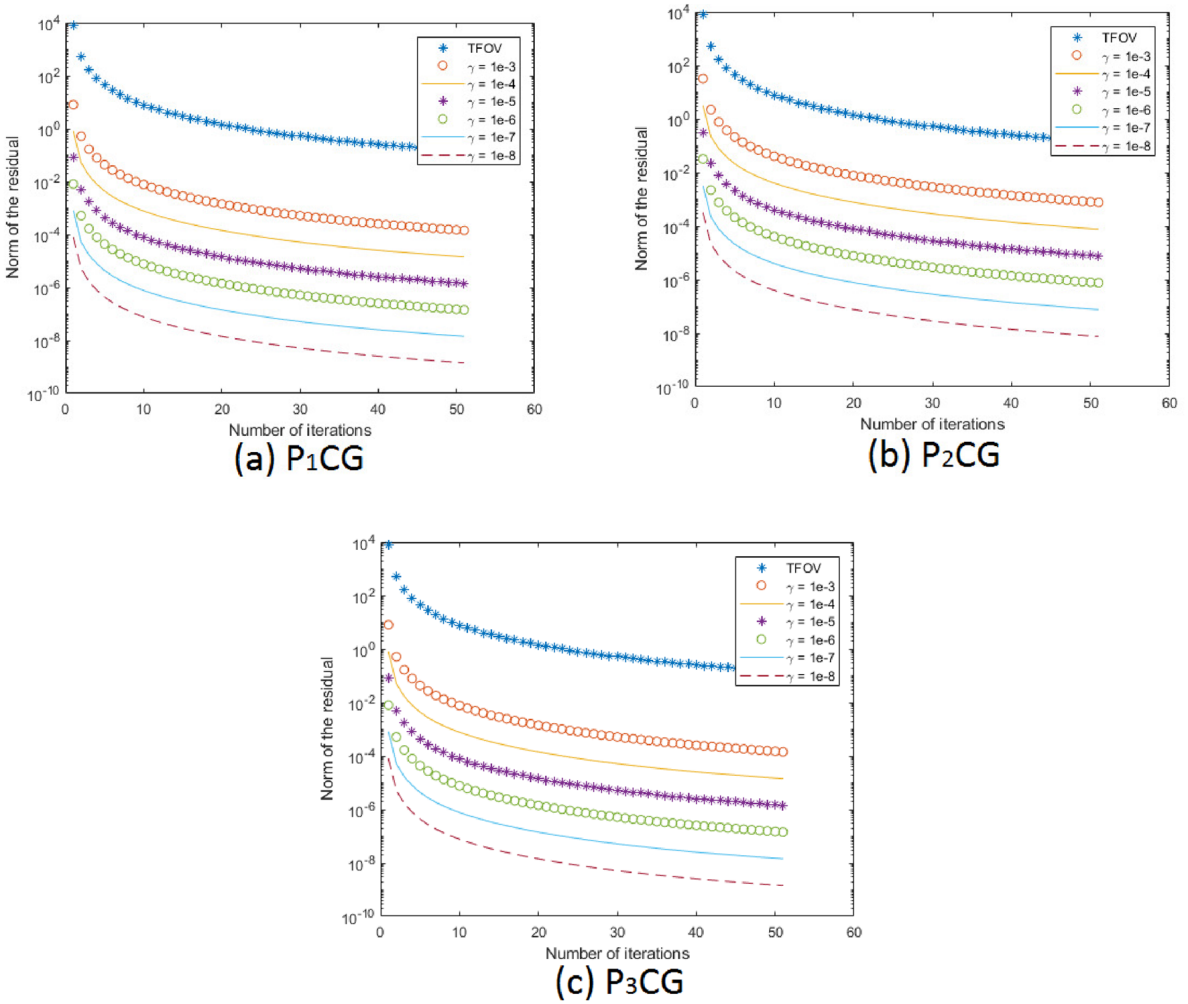
TFOV-based CG and PCG convergence at fixed point iteration $m = 1$ for Example 1. Blue asterisk represents TFOV-based CG iterations. (**a**) $P_1$CG iteration against different values of $\gamma$, (**b**) $P_2$CG iteration against different values of $\gamma$ and (**c**) $P_3$CG iteration against different values of $\gamma$.

The cameraman image was utilized as an example in this study. It is a complex image consisting of both small-scale texture (peacoat) and large-scale cartoon (face) components. Figure 2 illustrates different aspects of the cameraman image, with each subfigure sized at $512 \times 512$. These subfigures include: (a) the exact and (b) blurry images; (c), (d), (e), and (f) the deblurred images using TFOV-based CG, $P_1$CG with $\gamma = 1e$-3, $P_2$CG with $\gamma = 1e$-4, and $P_3$CG with $\gamma = 1e$-5, respectively. Figure 3 illustrates the computation of the relative residual at each iteration with respect to $\gamma$. Numerical calculations were performed using the $ke_{-}gen (N, 300, 5)$ kernel. The blurry image of cameraman had a PSNR of 20.4727 and an SSIM of 0.0701. A tolerance value of $tol = 1e$-6 was employed as the stopping criterion for the numerical method.

**Table 3:** Comparison of TFOV-based CG and PCG for Example 1.

Method	$\gamma$	Deblurred PSNR	Deblurred SSIM	Iterations
TFOV (CG)		38.8061	0.6661	$50^{+}$
$P_1$CG	1e−3	38.8057	0.6661	$50^{+}$
	1e−4	38.0255	0.6603	$50^{+}$
	1e−5	37.9854	0.6325	50
	1e−6	37.5307	0.6456	21
	1e−7	36.9852	0.6356	10
	1e−8	37.1521	0.6364	4
$P_2$CG	1e−3	37.7026	0.6167	$50^{+}$
	1e−4	37.7052	0.6169	$50^{+}$
	1e−5	37.5909	0.6182	$50^{+}$
	1e−6	36.9195	0.6225	37
	1e−7	36.2562	0.6301	17
	1e−8	37.3301	0.6333	6
$P_3$CG	1e−3	37.5893	0.6629	$50^{+}$
	1e−4	37.9954	0.6312	$50^{+}$
	1e−5	38.7584	0.6669	50
	1e−6	37.5981	0.6446	24
	1e−7	34.1256	0.5898	10
	1e−8	32.1919	0.5398	3

#### Remark

1. One can notice that Fig. 2c–f, all are having the almost same quality. This means TFOV-based CG and PCG ($P_1$CG, $P_2$CG and $P_3$CG) methods are generating the same quality result.
2. The effectiveness of preconditioning can be clearly observed from Fig. 3. When using a fixed point iteration count of $m = 1$, it is evident that the number of iterations used by PCG is significantly fewer compared to CG in order to achieve the desired accuracy of $tol = 1e$-6.
3. Table 3 and Fig. 3 demonstrate that both TFOV-based CG and PCG methods yield similar PSNR and SSIM values. However, PCG achieves better PSNR and SSIM with considerably fewer iterations. The $P_1$CG method reaches its best PSNR and SSIM in just $50^{+}$ iterations with $\gamma = 1e{-}3$, whereas TFOV-based CG requires significantly more iterations to achieve comparable results. Similarly, the $P_2$CG algorithm achieves its best PSNR and SSIM in $40^{+}$ iterations with $\gamma = 1e{-}4$, and $P_3$CG achieves its best results in just 50 iterations with $\gamma = 1e{-}5$.
4. Figure 3 also indicates that PCG exhibits rapid convergence when $\gamma$ is close to the value of $\lambda = 1e{-}6$ for all preconditioners ($P_1$, $P_2$, and $P_3$). This observation aligns with the selection of the optimal $\gamma$, which falls within the range of $\lambda \le \gamma \le 1$ as determined by eigenvalue analysis (Section “Preconditioned matrices“). Although smaller values of $\gamma$ can further reduce the number of iterations, they also result in decreased deblurred image quality (PSNR and SSIM).
5. Additionally, it is noted that both $P_1$CG and $P_3$CG methods achieve slightly higher PSNR and SSIM compared to $P_2$CG. Thus, in this example, the performance of preconditioners $P_1$ and $P_3$ proves to be more effective than that of preconditioner $P_2$.

#### Example 2

**Figure 4:**
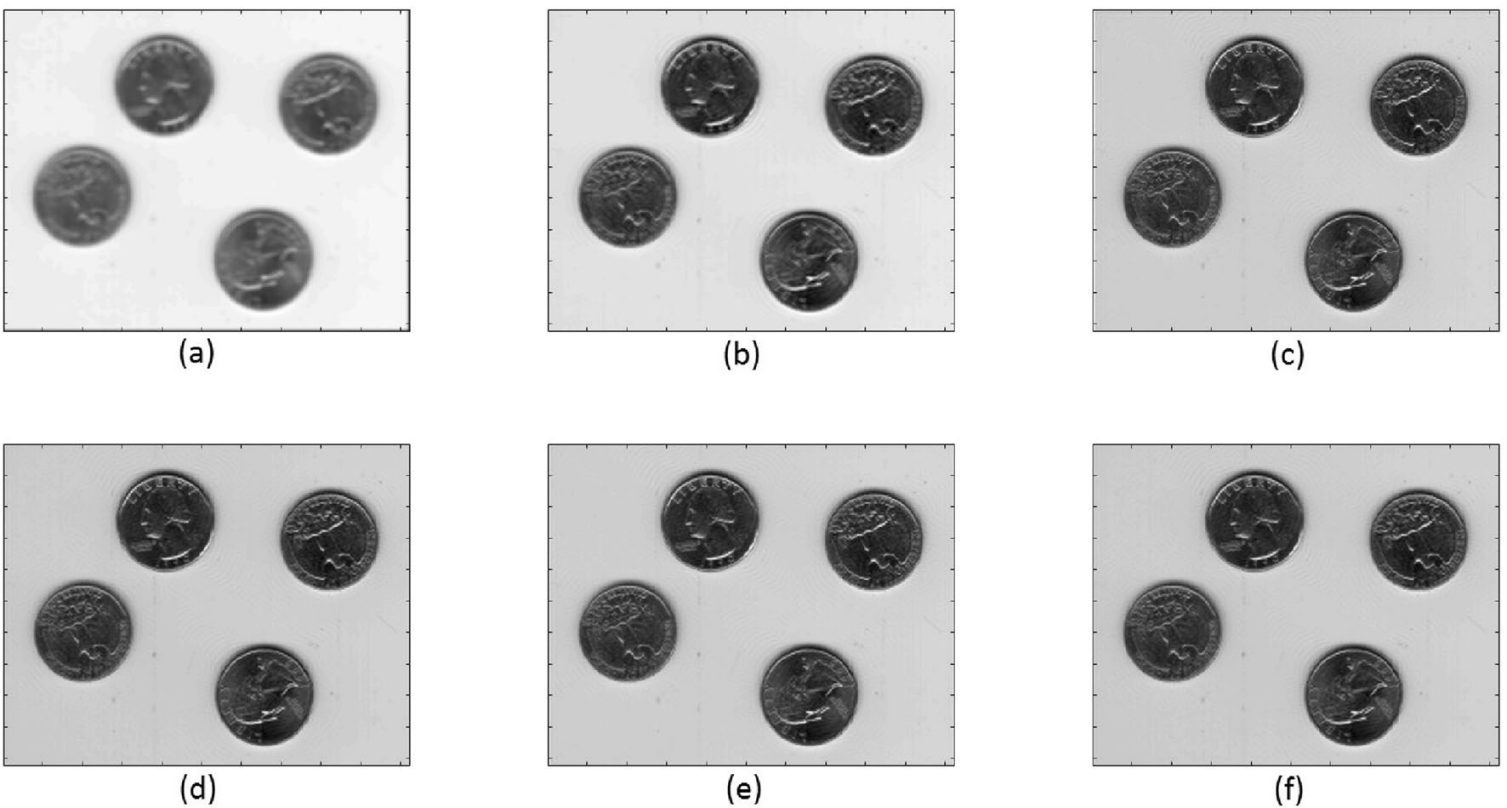
Coins image: (**a**) blurry image; (**b**–**f**) deblurred images by TV-based CG, TFOV-based CG, $P_1$CG, $P_2$CG, and $P_3$CG, respectively.

The Coins image, which consists of both real and synthetic components, was used in this example. A comparison was made between our TFOV-based algorithm and a TV-based method. Because the TV-based method generates a SPD matrix system, the CG (Conjugate Gradient) method was employed for its solution. Figure 4 presents different aspects of the Coins image, with each subfigure sized at $512 \times 512$. These subfigures include: (a) the blurry image; (b), (c), (d), (e), and (f) the deblurred images using TV-based CG, TFOV-based CG, $P_1$CG, $P_2$CG, and $P_3$CG, respectively. Numerical calculations were performed using the $ke_{-}gen (N, 300, 2)$ kernel. For the TV-based CG method, $\lambda = 5e - 4$ and $\beta = 1$ were used according to^10^. The values of $\gamma$ were set to $1e{-}3$ for $P_1$CG, $1e{-}5$ for $P_2$CG, and $1e{-}4$ for $P_3$CG. The stopping criterion for the numerical methods was set to a tolerance of $tol = 1e$-3.

#### Remark

**Figure 5:**
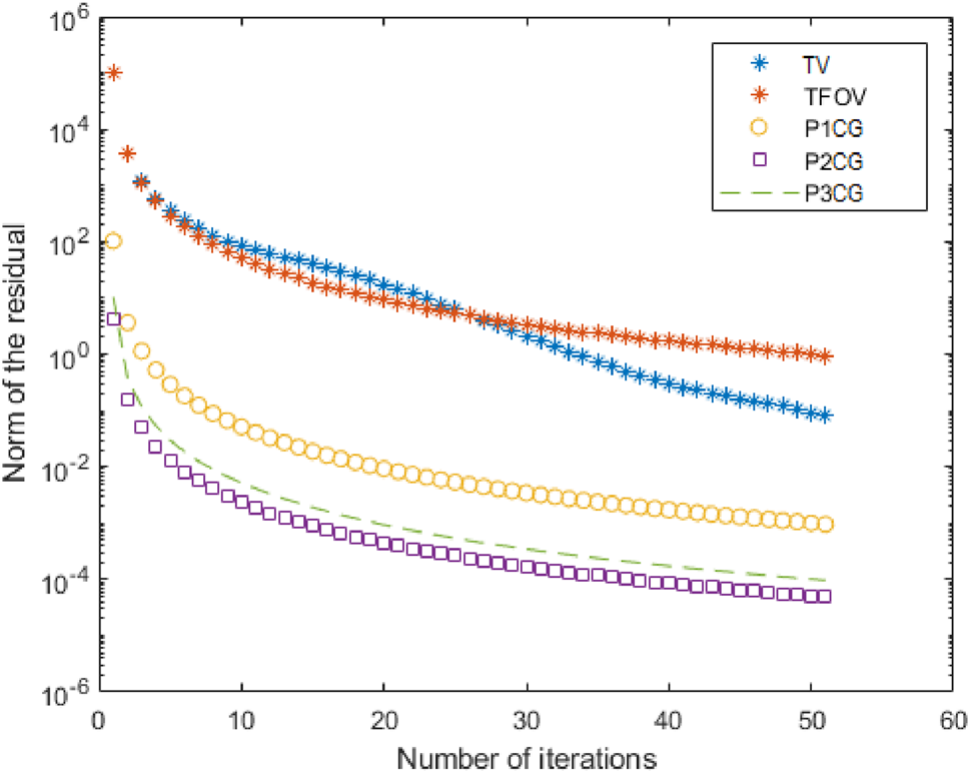
The TV-based CG, TFOV-based CG and PCG convergence at fixed point iteration $m = 1$ for Example 2. Blue asterisk represents TV-based CG iterations, red asterisk represents TFOV-based CG iterations, yellow circle represents $P_1$CG iteration, purple box represents $P_2$CG iteration and green line represents $P_3$CG iteration.

1. The comparison presented in Table 4 indicates that the TFOV-based methods (CG and PCG) achieve slightly higher PSNR and SSIM metrics compared to the TV-based CG method. This observation is further supported by Fig. 4b–f. Hence, the TFOV-based methods (CG and PCG) generate superior image quality results.
2. The effectiveness of preconditioning can be clearly observed from Fig. 5. The number of iterations required by TFOV-based PCG is significantly fewer compared to both TFOV- and TV-based CG methods in order to achieve the desired accuracy of $tol = 1e$-3.
3. Table 4 shows that the TFOV-based PCG method achieves slightly higher PSNR and SSIM values compared to the regular TFOV-based CG method. However, the PCG method achieves its PSNR and SSIM in significantly fewer iterations. The $P_1$CG method requires only 50 iterations, the $P_2$CG method requires only 14 iterations, and the $P_3$CG method requires only 18 iterations to achieve the desired results. In contrast, the TV-based CG method and the TFOV-based CG method both require more than 50 iterations to reach their respective PSNR and SSIM values. This demonstrates that the TFOV-based PCG algorithm is faster than both the TFOV-based CG method and the TV-based CG algorithm for real and synthetic images. Additionally, the effectiveness of preconditioner $P_2$ surpasses that of preconditioners $P_1$ and $P_3$ (Fig. 5).

**Table 4:** Comparison of TV-based CG, TFOV-based CG and PCG for Example 2.

	TV	TFOV			
	CG	CG	$P_1$CG	$P_2$CG	$P_3$CG
Blurred PSNR	23.9659	23.9659	23.9659	23.9659	23.9659
Blurred SSIM	0.3292	0.3292	0.3292	0.3292	0.3292
Deblurred PSNR	32.6141	35.2594	35.2635	35.5271	35.3002
Deblurred SSIM	0.5709	0.6068	0.6069	0.6109	0.6071
Iterations	$50+$	$50+$	50	14	18

#### Example 3

**Figure 6:**
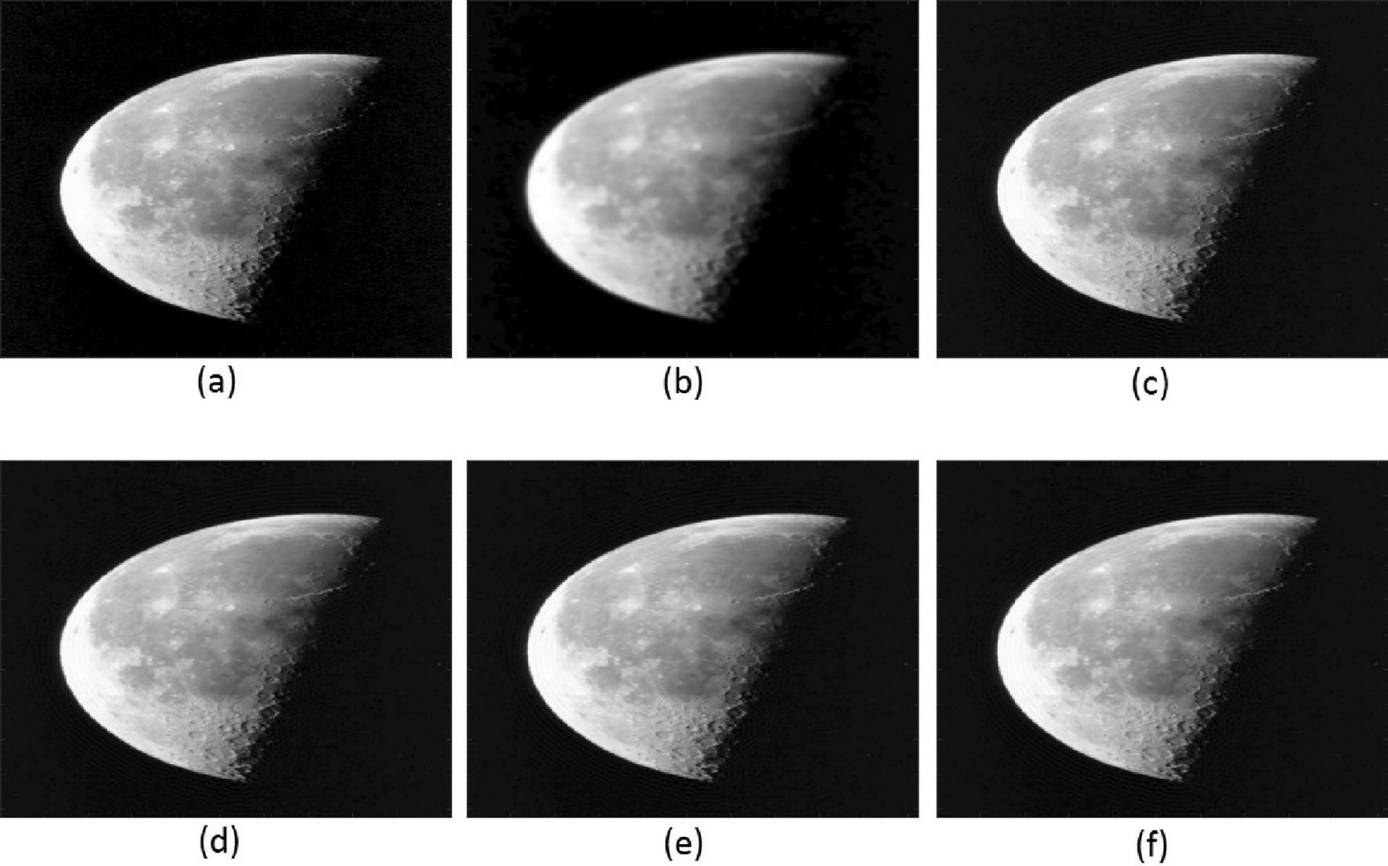
Moon image: (**a**) exact, (**b**) blurry; (**c**–**f**) deblurred images by TFOV-based CG, $P_1$CG, $P_2$CG, and $P_3$CG, respectively.

Here, we have utilized a nontexture Moon image. The various aspects of the Moon image are illustrated in Fig. 6. Each subfigure has a size of $512 \times 512$. They represent: (a) the exact, (b) blurry; (c), (d), (e) and (f) deblurred images using TFOV-based CG, $P_1$CG, $P_2$CG, and $P_3$CG, respectively. The numerical calculations were performed using the $ke_{-}gen(N, 300, 3)$ kernel. To facilitate comparison, we considered three different values of *N*: 128, 256, and 512. The stopping criteria for the numerical methods was set to a tolerance of $tol = 1e$-4.

#### Remark

**Figure 7:**
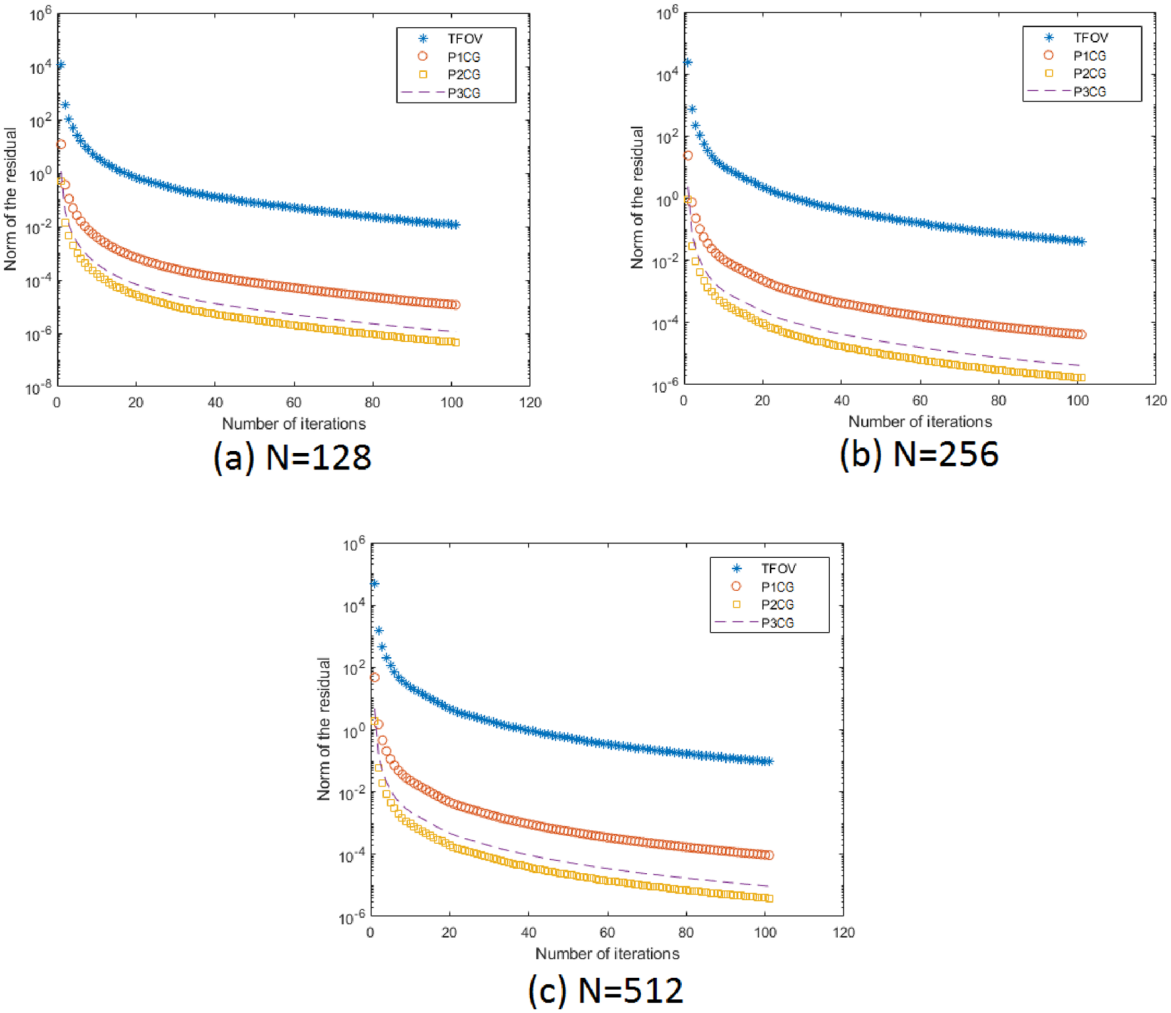
The TFOV-based CG and PCG convergence at fixed point iteration $m = 1$ for Example 3. Blue asterisk represents TFOV-based CG iterations, red circle represents $P_1$CG iteration, yellow box represents $P_2$CG iteration and black line represents $P_3$CG iteration.

1. The similarity between Fig. 6c–f indicates that all methods produce results of the same quality.
2. Figure 7 clearly shows that for all values of *N*, the number of iterations required by PCG ($P_1$CG, $P_2$CG, and $P_3$CG) is significantly fewer compared to TFOV-based CG in order to achieve the desired accuracy of $tol = 1e$-4.
3. Table 5 demonstrates that the PSNR and SSIM values obtained by the PCG method are almost identical to those achieved by the regular TFOV-based CG method for all values of *N*. However, the PCG method achieves these PSNR and SSIM values in significantly fewer iterations. For instance, for $N = 64$, the $P_1$CG method requires only 40 iterations, the $P_2$CG method requires only 10 iterations, and the $P_3$CG method requires only 19 iterations to attain the desired PSNR and SSIM values. In contrast, the CG algorithm requires over 100 iterations to achieve the same results. Similar observations can be made for other values of *N*. Thus, the PCG algorithm is faster than TFOV-based CG for nontexture images. Furthermore, the performance of all preconditioners is nearly the same (Fig. 7).

**Table 5:** Comparison of TFOV-based CG and PCG for Example 3.

Mesh size *h*	Blurred PSNR	Blurred SSIM	Method	Deblurred PSNR	Deblurred SSIM	Iterations
1/64	27.0862	0.5787	TFOV (CG)	54.6985	0.9568	$100+$
			$P_1$CG	54.6985	0.9568	40
			$P_2$CG	54.6542	0.9537	10
			$P_3$CG	54.6985	0.9568	19
1/128	28.9698	0.3768	TFOV (CG)	47.1319	0.8358	$100+$
			$P_1$CG	47.1319	0.8358	60
			$P_2$CG	47.1312	0.8350	18
			$P_32$CG	47.1319	0.8358	21
1/256	29.9274	0.1951	TFOV (CG)	45.2063	0.7948	$100+$
			$P_1$CG	45.2043	0.7947	100
			$P_2$CG	45.1638	0.7939	26
			$P_3$CG	45.1868	0.7943	40

#### Example 4

**Figure 8:**
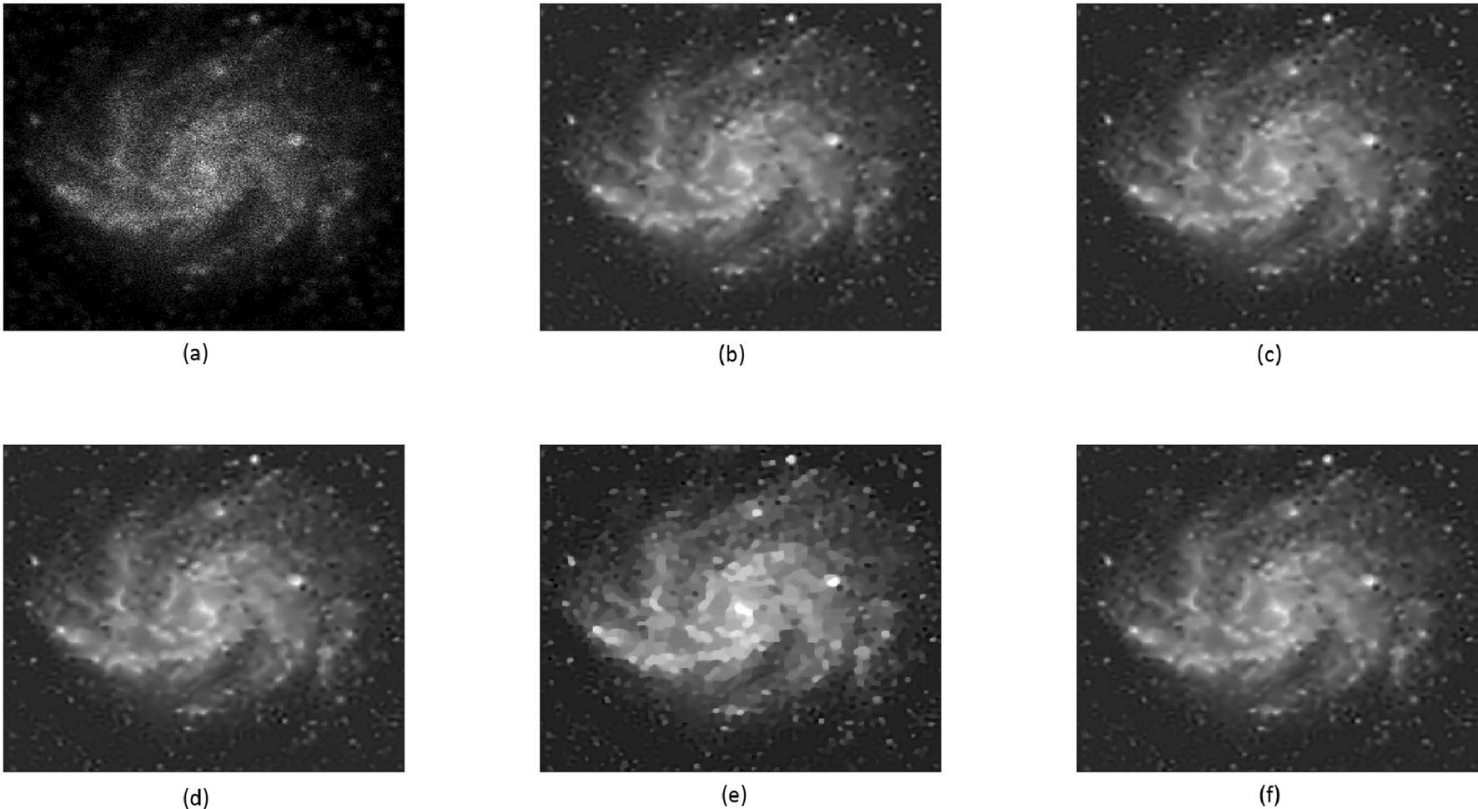
Galaxy image: (**a**) is blurry image; (**b**–**f**) are deblurred images by NFOV-based CG, TFOV-based CG, $P_1$CG, $P_2$CG, and $P_3$CG, respectively.

**Figure 9:**
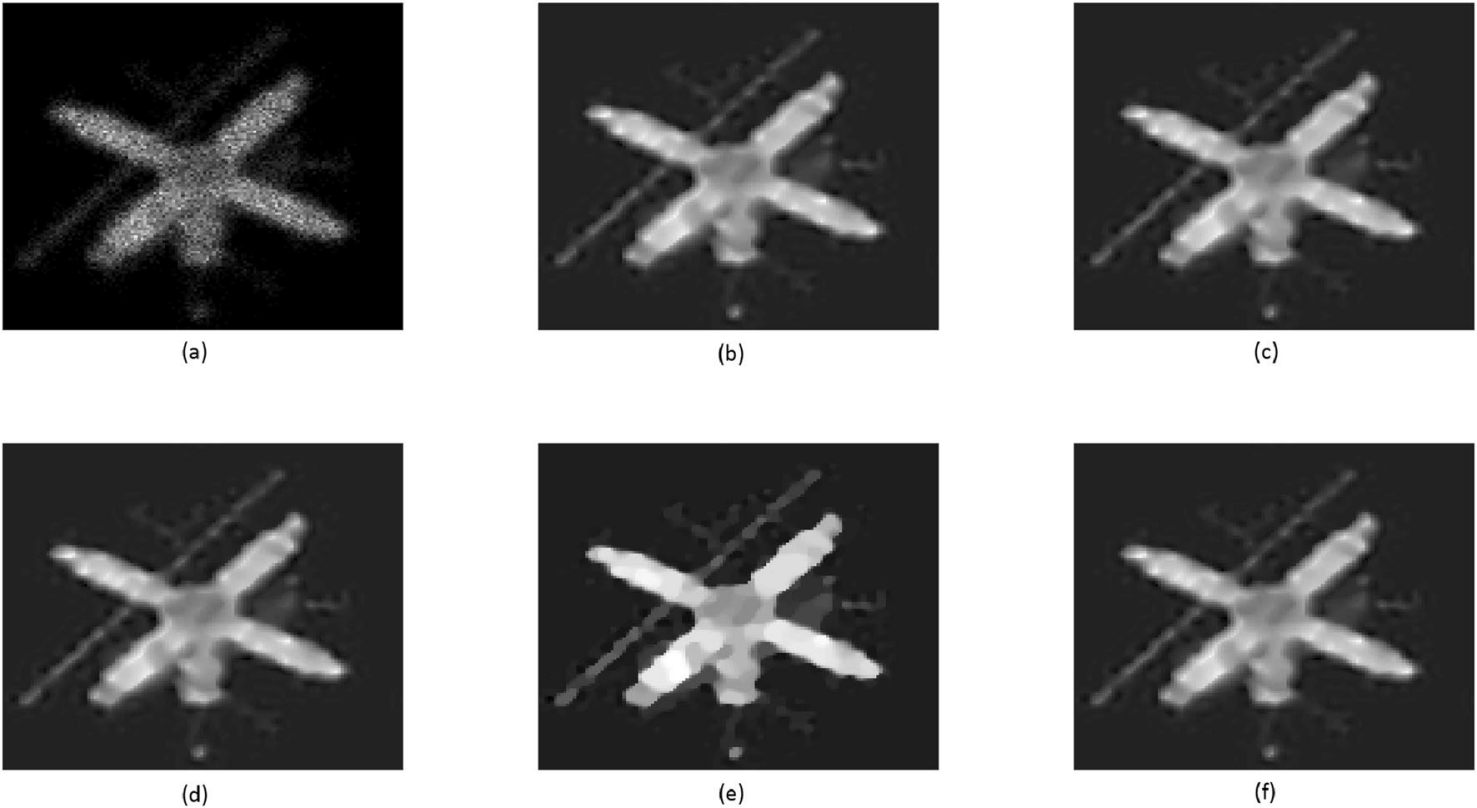
Satel image: (**a**) is blurry image; (**b**–**f**) are deblurred images by NFOV-based CG, TFOV-based CG, $P_1$CG, $P_2$CG, and $P_3$CG, respectively.

In this example, a pair of satellite images from^49^ were employed. These images were intentionally subjected to blurring and corruption by Poisson noise, leading to the presence of blurring artifacts. For the blurring process, we utilized a kernel with parameters $fspecial('gaussian',9,sqrt(3))$. The introduction of Poisson noise to the images poses a significant challenge for the majority of deblurring techniques. This type of noise commonly arises in scenarios involving photon counting within various imaging modalities. At the same time, blurring is an inevitable outcome due to the underlying physical principles of the imaging system, which can be conceptualized as the convolution of the image with a point spread function. For the purpose of comparison, we opted to utilize the approach by Chaudhury et al.^49^, known as the non-blind fractional order TV-based algorithm (NFOV). The restored images of the Galaxy can be observed in Fig. 8, each possessing dimensions of $256 \times 256$. Similarly, the restored images of Satel are depicted in Fig. 9, each sized at $128 \times 128$. For the NFOV method, we configured the parameters as outlined in^49^. The stopping criterion for the computational technique is determined by a tolerance value of $tol = 1e$-7. Further details regarding this experiment can be found in Table 6.

#### Remark

By referring to Figs. 8, 9, and Table 6, it becomes evident that the outcomes produced by all techniques are nearly indistinguishable. However, our suggested PCG methods yield slightly superior PSNR values while requiring significantly less CPU time. This observation highlights the enhanced effectiveness and swiftness of our proposed PCG methods compared to the NFOV method.

**Table 6:** Comparison of various algorithms for Example 4: PSNR, SSIM, and CPU-Time.

	Galaxy			Satel		
	PSNR	SSIM	CPU-Time	PSNR	SSIM	CPU-Time
Blurred	20.6620	0.6712	–	20.4559	0.7994	–
NFOV	24.1417	0.8222	90.1267	22.7439	0.8759	83.2569
TFOV (CG)	25.1592	0.8423	91.2549	24.3652	0.8856	83.5556
$P_1$CG	25.1642	0.8431	63.1486	24.3669	0.8857	60.5328
$P_2$CG	25.2036	0.8436	50.2614	24.3711	0.8884	51.2563
$P_3$CG	25.1931	0.8535	51.0243	24.3693	0.8861	50.8642

## Conclusion

We presented a numerical method (PCG) for solving the primal form of the total fractional order variation (TFOV) based nonlinear image deblurring problem. We introduced three novel circulant preconditioned matrices ($P_1, P_2$, and $P_3$) and tested them with three examples using PCG. Various types of images (real, complicated, non-texture, synthetic and satellite) were also tested using our new circulant preconditioned matrices. Additionally, we compared the TFOV-based algorithm (CG and PCG) with the TV (total variation) based algorithm and NFOV method^49^. The convergence rates and residual norms at each iteration were provided for each example. The numerical tests we conducted highlight the swift convergence achieved by the PCG method when employing the novel circulant preconditioners. Beyond the accelerated speed, the PCG method also exhibits efficacy in addressing the TFOV-based nonlinear image deblurring challenge. For this study, the images under consideration were grayscale; however, we anticipate extending our approach to encompass color images in future research. Additionally, we intend to apply our proposed method to images characterized by varying degrees of blurriness. Moreover, the crucial role in the deblurring process is also attributed to the ideal settings of parameters such as $\alpha$ and $\beta$. In the present study, we empirically analyze these parameters, while we intend to establish their precise theoretical constraints in subsequent research.

## Data Availability

The data used to support the findings of this study are available from the corresponding author upon request.
